# Association of Overweight and Consistent Anovulation among Infertile Women with Regular Menstrual Cycle: A Case-control Study

**DOI:** 10.1055/s-0041-1739464

**Published:** 2021-12-06

**Authors:** Christiane Ricaldoni Giviziez, Eliane Gouveia de Morais Sanchez, Yanna Andressa Ramos de Lima, Mário Silva Approbato

**Affiliations:** 1 Unidade Acadêmica de Ciências da Saúde, Universidade Federal de Jataí, Jataí, GO, Brazil; 2 Departamento de Ginecologia e Obstetrícia, Faculdade de Medicina, Hospital das Clínicas, Laboratório de Reprodução Humana, Universidade Federal de Goiás, Goiânia, GO, Brazil

**Keywords:** female infertility, anovulation, overweight, assisted reproductive techniques, infertilidade feminina, anovulação, sobrepeso, técnicas de reprodução assistida

## Abstract

**Objective** It has been suggested that excess body weight could represent a risk factor for infertility outcomes. The present study aimed to evaluate the association of overweight and anovulation among infertile women with regular menstrual cycles.

**Methods** We conducted a retrospective case-control study with consistently anovulatory patients undergoing assisted reproduction treatment. The patients were stratified into normal weight (body mass index [BMI]: 18.5–24.9kg/m ^2^ ) and overweight (BMI: 25.0–29.9kg/m ^2^ ).Those with polycystic ovary syndrome or obesity were excluded. The groups were matched for age, duration of infertility, prolactin, follicle stimulating hormone (FSH), thydroid stimulating hormone (TSH), luteinizing hormone (LH), and estradiol levels.

**Results** Overweight was significantly associated with anovulation, when using the World Health Organization (WHO) criteria for anovulation: progesterone levels > 5.65 ng/ml and ultrasonography evidence of follicle collapse (odds ratio [OR]: 2.69; 95% confidence interval [CI95%]: 1.04–6.98).

**Conclusion** Body mass index above the normal range jeopardizes ovulation among non-obese infertile women with regular menstrual cycles.

## Resumo

**Objetivo** O excesso de peso corporal tem sido associado como fator de risco para infertilidade. Este estudo teve como objetivo avaliar a associação de sobrepeso e anovulação entre mulheres inférteis com ciclos menstruais regulares.

**Métodos** Realizamos um estudo retrospectivo de caso-controle com mulheres com anovulação consistente em tratamento por reprodução assistida. As pacientes foram estratificadas entre aquelas com peso normal (índice de massa corporal [IMC]: 18,5–24,9 Kg/m ^2^ ) e as com sobrepeso (IMC: 25,0–29,9 Kg/m ^2^ ). As pacientes com síndrome do ovário policístico ou obesidade foram excluídas. Os grupos foram pareados por idade, duração da infertilidade, níveis de prolactina, hormônio folículo-estimulante (FSH), hormônio tiroestimulante (TSH), hormônio luteinizante (LH) e estradiol.

**Resultados** O excesso de peso associou-se significativamente à anovulaçãoquando usados os critérios de anovulação da Organização Mundial de Saúde (OMS): níveis de progesterona > 5,65 ng/ml e evidência ultrassonográfica de colapso folicular (razão de chances [RC]: 2,69; IC95%: 1,04–6,98).

**Conclusão** O IMC acima da faixa normal compromete a ovulação em mulheres inférteis não obesas com ciclos menstruais regulares.

## Introduction

Body mass index (BMI) is associated with risk for diabetes mellitus, arterial hypertension, obstructive sleep apnea, cancer, dyslipidemia, and cardiovascular disease. 1 Puberty, ovulation and reproductive function are biological events highly dependent on body energetic reserves. In this regard, variables such as weight, body composition, body fat distribution, and the effects of diet and physical exercise have been investigated in women with alterations in sexual maturation, menstrual cycle, and fertility. 2 3 4

Extremes in body size, such as thinness and obesity, are considered risk factors for ovulatory infertility among women. 5 Excess body weight is also associated with disorders of the menstrual cycle and infertility, especially ovulation disorders. 6 Infertile women with overweight present higher rates of oligomenorrhea, amenorrhea, hirsutism, and hormonal changes than infertile women with a normal BMI. 7 Ovulatory disorders represent 25 to 50% of all causes of infertility in women, and a significant proportion is directly or indirectly related to overweight and obesity. 8

The negative effects of female obesity on reproduction are associated with increased risk of miscarriage, decrease in pregnancy rate, worse results in assisted reproduction treatments, and lower rates of live births. 9 10 11 12 However, previous studies demonstrate controversial effects of high BMI, especially with regard to the results of assisted reproduction treatment. 13 These differences can be explained by the clinical, methodological and statistical heterogeneity of the studies, as well as the differences found in the definition of a normal BMI characteristic of overweight and obesity. 14

Therefore, the present study aimed to evaluate the association of overweight and ovulation profile among infertile women searching for assisted reproduction treatment with regular menstrual cycles and not diagnosed with polycystic ovary syndrome or obesity.

## Methods

A retrospective case-control study was conducted to evaluate the association of overweight and ovulation profile among patients with regular menstrual cycles.The current study was submitted and approved by the ethical review board of the university hospital of Universidade Federal de Goiás (protocol n°751,390), where the recruitment of patients was performed.

This retrospective study was performed at the Human Reproduction Laboratory of the University Hospital in Goiás, Midwest Brazil (LabRep). LabRep is a public health service that provides low cost assisted reproduction treatment for infertile patients.

We evaluated 7,200 medical records of female patients searching for assisted reproduction treatment between January 2004 and December 2014. According to the inclusion criteria, patients with regular menstrual cycle (25–35 days), aged between 20 and 40 years, and with BMI between 18.5 and 29.9 kg/m ^2^ (normal and overweight) were selected. Patients were excluded when presenting previous diagnosis of polycystic ovary syndrome (PCOS), as established by the Rotterdam consensus on diagnostic criteria (20); BMI > 29.9 kg/m ^2^ (obesity); follicle-stimulating hormone (FSH) > 9.9 nUI/mL; thyroid-stimulating hormone (TSH) between 0.4–5.0 mIU/mL; serum prolactin level > 20 ng/mL; self-reported diabetes; history of endometriosis, ovarian tumors, or use of medicines that interfere with ovulation.

The case and control groups were defined by the ovulation outcome, defined as consistent non-ovulatory (anovulation) or consistent ovulatory (ovulation), respectively. The exposure variable was overweight, defined by BMI above the normal range and below the obesity range.

Body mass index was calculated according to the Quetelet formula, defined as the weight in kilograms divided by the square of the height in meters (Kg/m ^2^ ). Patient's weight and height were evaluated at the time of the first medical attendance by an experienced nurse. Normal weight was considered when the patient's BMI was between 18.50 and 24.99 kg/m ^2^ , and overweight was considered when the patient's BMI was between 25.00 and 29.99 kg/m ^2^ .

Consistent ovulation or anovulation was defined by the agreement of two parameters: ultrasound monitoring of ovarian follicles and serum progesterone levels dosed 1 week after follicular collapse. Ultrasound monitoring of ovulation was performed between the 2 ^nd^ and 5 ^th^ day of the menstrual cycle, re-examined between the 9 ^th^ and 10 ^th^ day and on alternate days until ovulation or until the 16 ^th^ day, in cases with no development of dominant follicles or follicular collapse. Examinations were performed by an experienced medical doctor.

According to the World Health Organization (WHO), serum progesterone levels higher than 5.65 ng/mL are suggestive of ovulation. However, progesterone levels higher than 10 ng/mL are considered a more reliable threshold to predict ovulatory function according to the American Society for Reproductive Medicine (ASRM). 15

To further evaluate the ovulatory function in this set of patients, we used two thresholds for serum progesterone levels indicative of ovulation. We first considered as consistently ovulatory those patients with serum progesterone levels > 5.65 ng/mL (according to WHO) and with follicle collapse observed at ultrasonography (USG). In a second moment, we considered the progesterone levels ≥ 10.00 ng/mL (according to the ASRM) as indicative of ovulation, in association with USG evidence. Anovulation was considered when progesterone levels were ≤ 5.65 ng/mL or < 10.0 ng/mL with an associated absence of follicle collapse observed at USG.

Additionally, to compare serum progesterone levels among women with normal weight and progressive BMI ranges, the patients were stratified within four categories according to BMI: range I (normal weight with BMI 18.5–22.9 kg/m ^2^ ); range II (normal weight with BMI 23.0–24.9 kg/m ^2^ ); range III (overweight with BMI 25.0–27.5 kg/m ^2^ ); and range IV (overweight with BMI 27.5–29.9 kg/m ^2^ ). Medians of serum progesterone levels were obtained for each category.

The matching criteria used for case an control groups included: age (years), infertility duration (months), and serum levels of prolactin (ng/mL), FSH (ng/mL), luteinizing hormone (LH) (mIU/mL), TSH (mIU/mL), and estradiol (pg/mL). Hormonal levels were obtained as part of the routine service between the 2 ^nd^ and 4 ^th^ day of the menstrual cycle.

The statistical difference between independent samples was evaluated by the non-parametric Wilcoxon Mann-Whitney test. To evaluate the differences between proportions, we used either the Chi-squared or the Fisher exact test. The significance level was 5%. Statistical analyzes were performed with the support of IBM SPSS Statistis for Windows, Version 20.0 (IBM Corp., Armonk, NY, USA).

## Results

A total of 7,200 medical records of infertile female patients who underwent assisted reproduction treatment were retrieved from LabRep, a human reproduction public health service. Based on the inclusion and exclusion criteria, a total of 263 medical records of patients with regular menstrual cycles were selected. The mean age of these patients was 31.9 years (SD: ± 4. 4), ranging from 20 to 40 years. A total of 124 out of 263 (47%) were classified as having primary infertility and 139 out of 263 (53%) presented secondary infertility. The mean duration of infertility was 70.2 months (SD: ± 48.8).

The case and control groups were stratified according to the presence of the exposure variable (overweight) and matched. Table 1 presents possible confounding variables that were analyzed among patients with normal weight and those with overweight. The studied groups were comparable for all the analyzed variables.

**Table 1:** Clinical variables of infertile women with regular cycles with normal weight and overweight

Variables	Normal weight BMI 18.5–24.9 kg/m ^2^			Overweight BMI 25.0–29.9 kg/m ^2^			*P* -value
	n	Mean	SD	n	Mean	SD	
Age (years)	168	31.48	4.45	95	32.26	4.27	0.163
Infertility duration (months)	168	65.85	46.55	95	72.60	45.82	0.218
Prolactin (ng/mL)	167	11.14	4.01	95	10.23	3.74	0.058
FSH (ng/mL)	166	6.08	1.54	93	5.92	1.56	0.350
LH (UI/L)	155	4.39	2.02	89	4.06	1.98	0.201
TSH (mUI/L)	166	1.94	0.95	91	1.87	0.91	0.592
Estradiol (ng/dL)	157	51.11	27.09	87	48.67	30.34	0.197

Abbreviations: BMI, body mass index; FSH, follicle-stimulating hormone; LH, luteinizing hormone; SD, standard deviation; TSH, thyroid-stimulating hormone.

**Fig. 1:**
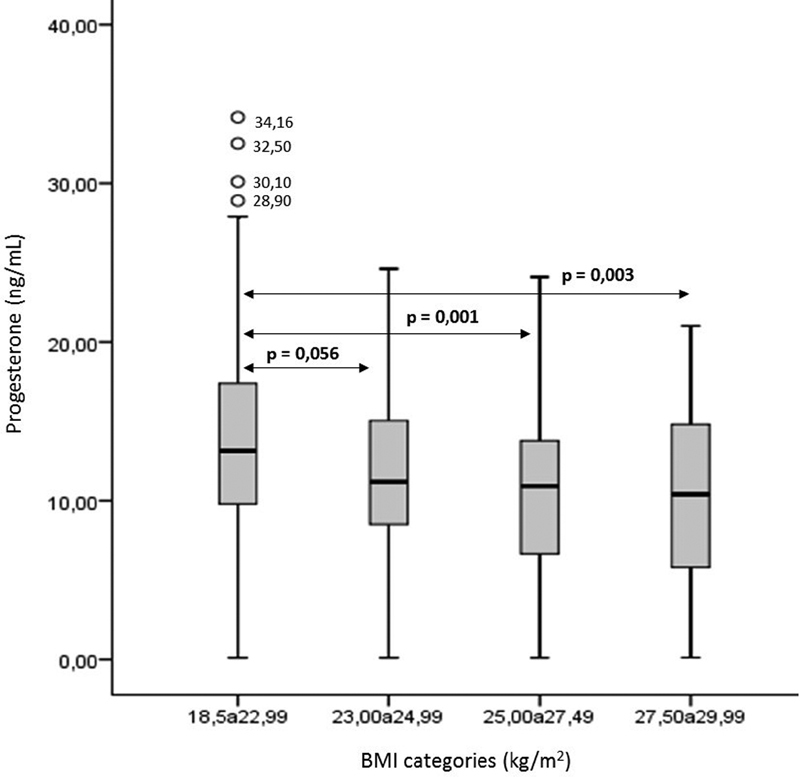
Median of serum progesterone level among patients within different BMI categories: 18.50 to 22.99 kg/m ^2^ (Range I: normal weight); 23.00 to 24.99 kg/m ^2^ (Range II: normal weight); 25.00 to 27.49 kg/m ^2^ (Range III: overweight) and 27.50 to 29.99 kg/m ^2^ (Range IV: overweight).

Figure 1 demonstrates that medians of serum progesterone gradually decrease as the weight of patients increase. Patients with normal weight presented significantly higher medians of serum progesterone than both ranges of overweight patients (ranges III and IV). In this way, we performed two analyses regarding ovulatory cutoff point based on serum progesterone levels, using both WHO and ASRM cutoffs indicative of ovulation (> 5.65 ng/mL and ≥ 10.0 ng/mL).

Among the 263 patients who met the inclusion criteria, 224 (85.2%) presented serum levels of progesterone > 5.65 ng/mL, and 168 (63.9%) presented serum levels ≥ 10.0 ng/mL, in accordance with the WHO and the ASRM criteria for ovulation, respectively. Regarding the USG evaluation, a total of 193 (73.4%) patients presented results indicative of ovulation. Patients were excluded if inconsistent ovulation was observed (serum progesterone level indicative of ovulation with no follicle collapse evidence by USG evaluation, or vice versa).

**Fig. 2:**
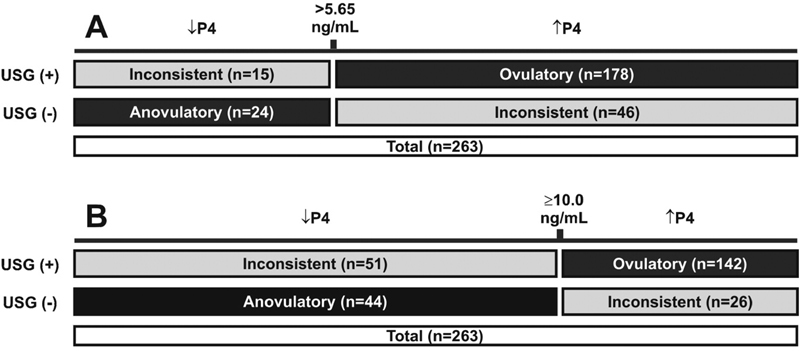
Characterization of infertile women as consistently ovulatory, anovulatory or inconsistently ovulatory using different cutoff points for serum progesterone levels and ultrasonography evidence of ovulation. A. Serum progesterone cutoff used for ovulation: > 5.65 ng/mL.B. Serum progesterone cutoff used for ovulation: ≥ 10.0 ng/mL.↓P4: low level of serum progesterone; ↑P4: high level of serum progesterone; USG (+): evidence of follicle collapse at ultrasonography; USG (-): lack of evidence of ovulation at ultrasonography.

Figure 2 demonstrates the distribution and characterization of patients as consistently ovulatory, anovulatory or inconsistently ovulatory using both cutoff points for serum progesterone levels.

When a serum progesterone level of 5.65 ng/mL was used as a cutoff point for ovulation, 61 out of 263 patients (23.2%) were excluded due to inconsistent ovulation. From the remaining 202 patients (76.8%) with consistently ovulatory or anovulatory status (confirmed with USG), 24 (11.9%) were anovulatory and 178 (88.1%) had a confirmed ovulation. Among the 178 consistently ovulatory patients, 117 (65.7%) presented normal BMI, and 61 (34.3%) were overweight. Regarding the 24 patients with consistent anovulation, 10 (41.7%) were within normal BMI range and 14 (58.3%) were considered overweight.

When using a cutoff point of ≥ 10 ng/mL for serum progesterone level, 77 out of 263 patients (29.3%) presented inconsistent ovulation and were excluded. A total of 186 patients was then analyzed: 44 (23.7%) characterized as consistently anovulatory and 142 (76.3%) characterized as consistently ovulatory. Among the 142 consistently ovulatory patients, 94 (66.2%) presented normal BMI and 48 (33.8%) were overweight. From the remaining 44 anovulatory patients, 23 (52.3%) were within normal BMI range and 21 (47.7%) were considered overweight.

Table 2 describes the association analyses for body weight and anovulation in this study group when using different thresholds for ovulation based on the serum progesterone level. Consistently anovulatory overweight women (characterized by serum progesterone ≤ 5.65 ng/mL and anovulation by ultrasound) were more than twice as likely to have anovulation when compared with those with normal weight (odds ratio [OR]: 2.69;CI 95%: 1.04–6.98; *p* : 0.022). However, no significant difference (OR: 1.79; CI95%: 0.79–4.10; p: 0.130) was observed among overweight women and those with normal weight diagnosed with consistently anovulation (characterized by serum progesterone < 10.0 ng/mL and anovulation by ultrasound).

**Table 2:** Distribution of infertile women with regular cycles according to consistent ovulation and overweight for both cutoff points of serum progesterone

BMI Categories (kg/m ^2^ )	Anovulatory n (%)	Ovulatory n (%)	*P* -value	Odds ratio (OR)	CI 95%
P4 > 5.65 ng/mL					
Overweight 25.0–29.9	14 (58.3)	61 (34.3)	0.022	2.69	1.04–6.98
Normal weight 18.5–24.9	10 (41.7)	117 (65.7)			
P4 ≥ 10.0 ng/mL					
Overweight 25.0–29.9	21 (47.7)	48 (33.8)	0.095	1.79	0.85–3.75
Normal weight 18.5–24.9	23 (52.3)	94 (66.2)			

Abbreviations: BMI, body mass index; CI, confidence interval; P4, serum progesterone.

## Discussion

In the present study, we observed that overweight increases more than twice the chance of anovulation among infertile women with regular menstrual cycles, with no previous diagnosis of polycystic ovary syndrome. This effect was observed when serum progesterone level > 5.65 ng/mL was used as an indication of ovulation. However, when progesterone levels ≤ 10.0 ng/mL and USG with no indication of follicular collapse were used as anovulation criteria, this difference was not observed. These findings corroborate the notion that excessive body weight contributes to infertility by affecting ovulation, probably by altering endocrine mechanisms. 6 16 17

The increase in body weight is the main determinant of insulinemia, insulin sensitivity, and ovarian hyperandrogenism. 18 Therefore, women who are overweight or obese may have altered endocrine profiles such as high LH, abnormal ratio of FSH and LH, low progesterone in the luteal phase, and low levels of sex hormone-binding globulin. 19

We observed that the serum progesterone levels gradually decreased as the weight of patients increased, and a significant difference in the serum levels of progesterone was observed between the normal weight and the overweight groups. This finding corroborates previous studies demonstrating that overweight women have low levels of serum progesterone during the luteal phase. 19 The use of progesterone levels alone for the diagnosis of ovulation is controversial, since the progesterone release is a pulsatile phenomenon and, therefore, varies during the day. However, serum progesterone level is commonly used as a markerfor ovulatory activity and measure of luteal function quality. 15 20

Regarding the weight classification, BMI is a marker routinely used for overweight and obesity. However, there is a lack of standardization of the indicative cutoff for overweight and obesity. Additionally, different BMI cutoff points for different ethnic groups should be considered as body fat percentage and distribution differ among populations.

Although previous studies demonstrated the association between anovulation and overweight, the high heterogeneity among them jeopardizes comparability. The present study provides important information for the assisted reproduction field as it demonstrates a significant association between overweight and anovulation among infertile patients with regular menstrual cycles and no diagnosis of obesity or polycystic ovary syndrome.

## Conclusion

We concluded that overweight can negatively influence the ovulatory profile of infertile women with regular cycles, when using the recommended criteria for ovulation by the WHO (serum progesterone level > 5.65 ng/ml and ultrasound evidence). However, no association was observed when the serum progesterone level threshold for ovulation was ≥ 10.0 ng/mL. Further studies are needed to evaluate different cutoff points for progesterone serum levels used as criteria for ovulation. Additionally, information regarding the frequency of anovulation among women with regular cycles and the impact of overweight in assisted reproduction treatments and pregnancy outcome should be further evaluated.
